# Metallo-Beta-Lactamase-Producing *Acinetobacter* spp. from Clinical Isolates at a Tertiary Care Hospital in Ghana

**DOI:** 10.1155/2020/3852419

**Published:** 2020-09-24

**Authors:** Michael A. Olu-Taiwo, Japheth A. Opintan, Francis Samuel Codjoe, Akua Obeng Forson

**Affiliations:** ^1^Department of Medical Laboratory Science, School of Biomedical and Allied Health Sciences, College of Health Sciences, University of Ghana, Legon Accra, Ghana; ^2^Department of Medical Microbiology, Ghana Medical School, College of Health Sciences, University of Ghana, Legon Accra, Ghana

## Abstract

Metallo-beta-lactamase-producing *Acinetobacter* spp. is a major challenge for therapeutic treatment of nosocomial infections. This study is aimed at determining the prevalence of MBL-producing *Acinetobacter* spp. among 87 clinical isolates of *Acinetobacter* spp. from the Korle-Bu Teaching Hospital, Accra, between August 2014 and July 2015. *Acinetobacter* spp. was identified by standard bacteriological method, and resistance to different antibiotics was assessed with the Kirby–Bauer disc diffusion method. Meropenem-resistant *Acinetobacter* isolates were screened for enzyme activity using the modified Hodge test (MHT) and combined disc test (CDT). Additionally, multiplex PCR was used to determine MBL genes presence (*bla*VIM_,_*bla*IMP, and *bla*NDM). All *Acinetobacter* isolates showed high resistance to cefotaxime (90.8%), ceftazidime (75.9%), cotrimoxazole (70.1%), ciprofloxacin (64.4%), gentamicin (72.4%), levofloxacin (67.8%), and meropenem (59.8%). A total of 54 (62.1%) of *Acinetobacter* isolates were multidrug-resistant. Out of 52 (59.8%) meropenem-resistant *Acinetobacter*, 3 (5.8%) were carbapenemase producers by MHT, whilst, 23 (44.2%) were CDT positive. There was no significant difference between the resistance pattern of amikacin, ceftazidime, cotrimoxazole, ciprofloxacin, and meropenem amongst CDT-positive and CDT-negative isolates (*p* > 0.05). A total of 7/87 (8.1%) CDT-positive *Acinetobacter* isolates harboured *bla*NDM; of these, 4 (57.1%) were from wound swabs, urine (*n* = 2) (28.6%), and ear swab (*n* = 1) (14.3%). The study revealed that less than 9% of *Acinetobacter* spp. contained *bla*NDM encoding genes. Strict antibiotics usage plan and infection control measures are required to prevent the spread of these resistance genes.

## 1. Introduction

In recent times, *Acinetobacter* spp. has been implicated in nosocomial infections of clinical importance in the elderly, infants, and immune-compromised patients [1–3]. Infections with *Acinetobacter* spp. lead to high mortality and morbidity, prolonged hospital stays with increased treatment costs. Carbapenem (meropenem and imipenem) are among the last resort for the treatment of serious Gram-negative bacilli infections [4]; however, carbapenem-resistant *Acinetobacter* has been on the increase to such an extent that carbapenem-resistant *A. baumannii* has been enlisted as one of the top priority pathogens by World Health Organization in 2017 [5]. Resistance to carbapenem could evolve by the development of efflux pumps, decreased cell permeability, and by the production of intrinsic or acquired carbapenemases belonging to either the class B or class D oxacillinases [6–8].

The emergence and dissemination of metallo-beta-lactamase- (MBL-) producing *Acinetobacter* spp. have become an important public health issue globally [4]. In Iraq, 56.6% of *Acinetobacter* spp. was reported to be phenotypic MBL producers, of which, 10.73% and 2.8% harboured *bla*IMP and *bla*VIM [9]. In Egypt, a recent study reported 86.7% of *A. baumannii* was phenotypic MBL producers, whilst a similar study in Egypt reported 34.6% phenotypic MBL in *A. baumannii*, 23.07% harboured *bla*VIM and *bla*IMP [10, 11]. In South Africa, 45% of *A. baumannii* was reported to be phenotypic MBL producers [12]. A previous study by Codjoe et al. [13] in Ghana reported a 14.4% prevalence for *bla*NDM in Gram-negative bacilli; however, the study's focus was on Gram-negative bacilli in general and not on the prevalence of MBL encoding genes in *Acinetobacter* spp. Limited information is available on carbapenemase activity in clinical isolates in Ghana; therefore, it is important to investigate MBL producers within the health-care settings, as early detection is critical for the effective formulation of antibiotic guidelines as well as the implementation of infection control strategies. This study is aimed at determining phenotypically and genotypically the prevalence of MBL encoding genes (blaIMP, *bla*VIM, and *bla*NDM) in carbapenem-resistant *Acinetobacter* spp. in a tertiary care hospital in the Accra metropolis.

## 2. Materials and Methods

### 2.1. Study Design and Site

This was a retrospective study of routinely collected clinical isolates of *Acinetobacter* spp. from Korle-Bu Teaching Hospital (KBTH). Korle-Bu Teaching Hospital (KBTH) is one of the largest health care facilities located in Accra, and it serves to provide health care for all categories of persons in Ghana [14]. It has more than 2000-bed capacity with intensive care units that cater for surgical, medical, and trauma emergencies. It serves a paediatric and adult population of over 3 million in the Greater Accra region and acts as a major referral health facility for an estimated population of 22 million people across Ghana [14].

### 2.2. *Acinetobacter* Isolates

This study used clinical isolates of the genus *Acinetobacter* spp. recovered consecutively from 16000 clinical samples that included aspirates, eye swabs, ear swabs, wound swabs, and urine submitted to the Central Microbiology Laboratory of KBTH from August 2014 to July 2015. A total of 87 nonduplicate *Acinetobacter* isolates identified as causative agents of infection were inoculated into brain heart infusion broth (Difco/BD Diagnostic systems, Sparks, Michigan, USA) supplemented with 30% glycerol and stored at -80°C freezer at the Department of Medical Microbiology, University of Ghana Medical School, for subsequent tests and analyses. The clinical isolates were identified as per standard bacteriological methods using Gram stain, Triple sugar iron (TSI) fermentation test, and Oxidase, Indole, and Citrate utilization test as described by [15].

### 2.3. Antimicrobial Susceptibility Test for *Acinetobacter* spp.

Isolates were subjected to Kirby–Bauer disc diffusion sensitivity testing per guidelines of the Clinical and Laboratory Standard Institute (CLSI) with Mueller-Hinton Agar [16] and EUCAST [17]. The following antibiotics purchased from Thermo Scientific™ Oxoid (United Kingdom) were used: amikacin (30 *μ*g), gentamicin (10 *μ*g), ampicillin (10 *μ*g), ciprofloxacin (5 *μ*g), levofloxacin (5 *μ*g), meropenem (10 *μ*g), amoxicillin/clavulanate (30 *μ*g), cefuroxime (30 *μ*g), ceftazidime (30 *μ*g), cotrimoxazole (25 *μ*g), and nitrofurantoin (300 *μ*g). *Acinetobacter* isolates with reduced susceptibility to meropenem (10 *μ*g) antibiotic disc (inhibition zone diameter ≤ 14 mm) were selected for carbapenemase and Metallo-beta-lactamase detection (modified Hodge test and combined disc test) as described by Lee et al. [18] and Yong et al. [19]. *Pseudomonas aeroginosa* ATCC 27853 and *Escherichia coli* ATCC 25922 were used as control strains for quality control. According to the international standard definition for acquired resistance, multidrug-resistant (MDR) phenotype was defined as in *vitro* nonsusceptibility to at least one agent in three or more categories of antimicrobials [20].

### 2.4. Phenotypic Carbapenemase Screening Methods

All the carbapenem-resistant *Acinetobacter* isolates were screened for carbapenemase activity by the modified Hodge and combined disc methods. The modified Hodge test (MHT) was performed as previously described by Lee et al. [18]. Briefly, a control strain of *Escherichia coli* (ATCC 25922) adjusted to 0.5 McFarland turbidity standard of inoculum was inoculated onto Mueller-Hinton (MH) agar (Oxoid, UK) as recommended by CLSI guidelines [16] and the plates were allowed to dry for 5 mins; then, one imipenem disc (10ug) (Oxoid, UK) was applied aseptically at the centre of the inoculated MH agar plate, and a 0.5 McFarland standard inoculum of *Acinetobacter* isolates was streaked from the edge of the imipenem disk (10ug) to the edge of the MH agar plate. After aerobic incubation at 37°C for 18-24 hrs,MH agar plates were observed for cloverleaf effect or indentation at the intersection of the test bacterium and the *E. coli* ATCC 25922 control strain within the inhibition zone of the imipenem disc (10 *μ*g) [18].

The combined disc test (CDT) was performed as previously described by Yong et al. [19]. Test organism of carbapenem-resistant *Acinetobacter* with a turbidity of 0.5 McFarland standard was inoculated onto Mueller-Hinton agar plate (Oxoid, UK) [16]. A 0.5M EDTA solution was prepared by dissolving 18.61 g of disodium EDTA in 100 ml of distilled water and adjusting its pH to 8.0 by the addition of NaOH and then autoclaved [19]. Two 10 *μ*g imipenem discs (Oxoid, UK) were placed onto the inoculated surface of the MH agar plate at 20 mm apart from center to center and 5 *μ*l of the prepared 0.5 M EDTA solution was added to one imipenem disc (Oxoid, UK) to obtain a desired concentration of 750 *μ*g. After 16-18 hrs of aerobic incubation at 37°C, the inhibition zone displayed around imipenem (Oxoid, UK) and imipenem-EDTA were read and compared. A zone size difference of ≥7 mm was taken as indicative of metallo-beta-lactamase production as described by Yong et al. [19]. This procedure was repeated to ensure the reproducibility of the result.

### 2.5. DNA Extraction of MBL Producers of *Acinetobacter* spp

Bacterial DNA template for PCR assay was extracted by using the whole-cell boiled lysate method [21]. Briefly, four to five colonies of fresh bacterial culture on MacConkey agar were transferred into 500ul of sterile saline in an Eppendorf tube and vortexed briefly, followed by heating at 100°C for 10 min. These were then centrifuged at 17 000 g for 5 min, and the supernatants were transferred into fresh Eppendorf tubes to serve as a DNA template for subsequent polymerase chain reaction (PCR).

### 2.6. PCR Detection of MBL Encoding Genes of *Acinetobacter* spp

Polymerase chain reaction for MBLs encoding genes was carried out using Eppendorf, master cycler epgradient S (BIORAD, USA). Briefly, multiplex PCR amplification was performed to detect MBL genes (*bla*IMP, *bla*VIM, and *bla*NDM) [22] (Table 1). Each reaction mixture contained 5 *μ*l of 4x PCR buffer, 4 mmol ^−1^ MgCl_2_, 2 *μ*l of 2 mmol deoxynucleotide triphosphate (dNTPs), 0·5 *μ*l of 25 pmol each of oligonucleotide primers (IMP-1, VIM-1, and NDM-1) and 1 U of Taq polymerase (Biolab, New England), and included 2 *μ*l DNA template. The volume was adjusted with sterile distilled water to give 25 *μ*l.

The amplification was carried out by an initial denaturation at 95°C for 5 min, followed by 35 cycles of denaturation at 94°C for 30 sec, annealing at 53°C for 30 sec, extension at 72°C for 1 min, and ending with final extension at 72°C for 7 min. The PCR products were analysed by electrophoresis using a 1·5% agarose gel, containing ethidium bromide. *Klebsiella pneumoniae* NCTC 13443 was used as *bla*NDM positive control, *Pseudomonas aeroginosa* NCTC 13437 as *bla*VIM positive control and nuclease free water was used as negative control.

### 2.7. Statistical Analysis

Data was entered into Microsoft Excel (2010) database and analyzed descriptively with SPSS version 20.0 (SPSS Inc., Chicago, IL). Frequency tables were used to display numbers, percentages of isolates, antibiotic resistance profiles and other variables. Chi-square (*X*^2^) was used for comparison of any two proportions or percentage; *p* value ≤ 0.05 was taken as statistically significant.

## 3. Results

### 3.1. *Acinetobacter* Isolates' Demographics and Characteristics

In the present study, out of 2950 Gram-negative bacilli isolated at the bacteriology unit of KBTH between August 2014 and July 2015, 3.0% (87) were *Acinetobacter* isolates. The isolates were from wound swabs [45 (51.7%)], urine [25 (28.7%)], ear swabs [8 (9.2%)], eye swabs 6 (6.9%), and aspirates [3 (3.5%)] (Table 2). The majority of these isolates were obtained from patients who were >50 years of age. Females (62.1%) had a high prevalence of *Acinetobacter* isolates compared to the males (37.9%) (Table 2).

### 3.2. Antibiotic Susceptibility Pattern of *Acinotobacter* Spp.

Based on the Kirby-Bauer disc diffusion test, 62.1% (54) of *Acinetobacter* isolates were multidrug-resistant.

High levels of resistance were observed for ceftazidime (75.9%), ciprofloxacin (64.4%), cefotaxime (90.8%), cotrimoxazole (70.1%), and meropenem (59.8%) (Table 3).

### 3.3. Resistance Patterns of CDT+ve and CDT-ve Isolates

The resistance patterns of CDT positive and CDT negative *Acinetobacter* isolates are shown in (Table 3). Relatively high levels of antibiotic resistance were observed in both combined disc test positive and combined disc test negative *Acinetobacter* isolates (*p* > 0.05) (Table 3). Amongst CDT positives, there was 100%, 100%, 100%, and 100% resistance to ampicillin, cefotaxime, cefuroxime, and ceftazidime, whilst CDT negatives isolates showed resistance of 93.1%, 93.1%, 93.1%, and 100%, respectively. Table 3.

### 3.4. Phenotypic-Based Carbapenemase Detection of *Acinetobacter spp.*

Phenotypic carbapenemase detection was observed in 5.8% (3/52) meropenem-resistant *Acinetobacter* by the modified Hodge test (MHT) and 44.2% (23/52) by the combined disc test (CDT). CDT-positive *Acinetobacter* isolates were predominant among patients who were >50years (39.12%), followed by 31-40 years (21.7%) and <1 years (13.0%). CDT-positive *Acinetobacter* isolates were predominant amongst female patients (65.2%) (Table 4). Between CDT-positive and CDT-negative isolates, gender distribution was not significant (*p* > 0.05) (Table 4). CDT-positive isolates were mostly obtained from cultures of urine, wound, and ear swabs. Among wound and urine isolates, the difference between CDT-positive and CDT-negative isolates were statistically significant (*p* < 0.05) (Table 4).

### 3.5. MBL Encoding Genes (*bla*NDM) among Specimen, Gender, and Age Group

Table 4 shows the distribution of MBL encoding genes in relation to the combined disc test. *bla*NDM genes were obtained relatively more in female, wound swabs, and among children <1 year-old, whilst, lesser *bla*NDM genes were obtained from male, urine specimen, and adults >50 years old.

## 4. Discussion

### 4.1. Antimicrobial Susceptibility Pattern of *Acinetobacter* spp.

The emergence of multidrug-resistant *Acinetobacter* isolates is a major concern in hospital settings in many parts of the world [4]. This present study found a high level of resistance to different classes of antibiotics among *Acinetobacter* isolates. Sixty-two percent (62%) of the isolates were resistant to at least three or more antimicrobial agents. This is consistent with previous reports from Italy (54%) and United States (72%) [23, 24]. However, in contrast to our findings, higher prevalence is reported in Sudan (97.0%) and India (91%) [25, 26]. The differences in prevalence may be associated with varying dependency on antibiotic usage in different countries [27].

Amongst third-generation cephalosporin in the present study, 75.9% of the isolates were resistant to ceftazidime. This is consistent with studies in China, India, and Egypt which reported resistance levels of 83.98% and 84.0% and 89.0%, respectively [11, 28, 29]. In contrast to the present study, a higher level of resistance to ceftazidime has been reported in Sudan (96%) and Benin (100%) [25, 30]. In this study, *Acinetobacter* resistance to cefotaxime was 90.8%. This is comparable to studies in Pakistan (99.2%) and India (100%) [25, 31]. The high level of resistance observed to the tested cephalosporins by *Acinetobacter* spp. may be due to a high level of extended-spectrum-*β*-lactamase (ESBLs) induced by selective pressure from broad-spectrum antimicrobial therapy [32].

In the present study, 64.4% of the isolates were resistant to ciprofloxacin. This is consistent with studies in India (64.0%) and South Africa (65%) [29, 32]. Whilst, higher prevalence are reported in Egypt (88.8%) and Brazil (80%) [11, 33], our findings are higher than studies from Benin (16%) and Nigeria (40.3%) [30, 34]. In the current study, 67.8% of *Acinetobacter* isolates were resistant to levofloxacin. In contrast to our finding, higher prevalence is reported in Mexico (78.1%) and China (82.5%) [28, 35]. In Ghana, the standard treatment guideline recommends the use of ciprofloxacin for the treatment of urinary tract and bloodstream infections [36]. The high level of resistance observed for fluoroquinolones in the present study is quite worrisome and may be due to inappropriate and irrational use of these antibiotics [37].

*Acinetobacter* isolates were 70.1% resistant to cotrimoxazole. In contrast to findings in the present study, in India, a slightly higher resistance of 89% was reported [38]. Resistance to gentamicin was found to be 72.4%. This is comparable with a study in Benin (75%) [30]. In contrast to this study, lower prevalence is reported in Cameroon (52.63%) and South Africa (58%) [32, 39]. The differences in prevalence may be due to antibiotic policy on the usage of gentamicin for empirical treatment.

Carbapenems have become one of the few selections for the treatment of *Acinetobacter* and other Gram-negative bacilli infections due to their wider spectrum of antibacterial activity and minimal side effects [4]. The prevalence rate of carbapenem resistance in *Acinetobacter baumannii* has been found to vary from one country to another [27, 40]. In the present study, 59.8% *Acinetobacter* isolates were resistant to carbapenem (meropenem). This is comparable with previous studies in Ghana (66%), Pakistan (58.9%) and Nigeria (63.6%) [13, 31, 41]. Whilst, studies by Hussein et al., [42] and Ren et al., [28] have reported carbapenem (imipenem) resistance of 58.26% in Iraq and 66% in China. Morfin-Otero *et al*., [43] and Rajput & Naik, [44] in Mexico and India have reported a lower carbapenem (imipenem) resistance prevalence of 48% and 48.57%, respectively. Furthermore, Fattouh and Nasr-Eldin, [10] in Egypt, have reported a higher carbapenem resistance of 71.4% to *Acinetobacter* spp. The high resistance to meropenem may be due to the intrinsic ability of *Acinetobacter* to quickly utilize the efflux pumping mechanism or the capacity to acquire resistant determinants from the environment in response to selective pressure [45].

### 4.2. Prevalence of MBL Producers in *Acinetobacter* spp

Among the 52 (59.8%) meropenem-resistant *Acinetobacter* isolates, less than 6% (3/52) were positive carbapenemase producers by the modified Hodge test (MHT); although, low sensitivity and specificity of the MHT method in the detection of MBL-producing isolates have been reported by several studies [7, 46]. The modified Hodge test is a first-line detection method for carbapenemase detection in carbapenem-resistant *Enterobacteriaceae* [47]. Since, there is no specific phenotypic method phenotypic method recommended for carbapenemase screening and confirmation in *Acinetobacter* spp. [47]. Different investigators, therefore, employ various detection methods like the combined disc test, double-disc synergy test, and MBL E-test [12, 18, 19].

The present study used the combined disc test (CDT) for phenotypic MBL detection; this method is specific, sensitive, and easy to perform in a different range of laboratories [19]. The phenotypic prevalence of CDT-positive *Acinetobacter* isolates was 44.2%. This is similar to studies in South Africa and India which reported a CDT prevalence of 45% and 44.8%, respectively [12, 48]. In contrast to findings in this study, a lower CDT prevalence of 34.3% was reported in Egypt [11]. However, in Iran, 86.8% of prevalence is reported [49]. The varying MBL prevalence may be due to the different phenotypic detection methods employed by the various investigators in the different countries [12, 18, 19].

### 4.3. Prevalence of Metallo-Beta-Lactamase Encoding Genes

Metallo-beta-lactamase-producing *Acinetobacter* infections have become a public health concern due to few therapeutic choices for the treatment of such infections. MBL-producing *Acinetobacter* possesses intrinsic potential to acquire and maintain resistance genotypes to different classes of antibiotics (beta-lactams and non-beta-lactams) [46]. Molecular-based techniques are the gold standard for the identification and differentiation of carbapenemase genes [21]. In the present study, multiplex polymerase chain reaction (PCR) was used to evaluate the presence of some MBL encoding genes (*bla*VIM, *bla*IMP, and *bla*NDM) [21]. Among the 23 (44.2%) CDT-positive *Acinetobacter* isolates, 7 (8.1%) haboured MBL encoding gene (*bla*NDM). However, no *bla*IMP and *bla*VIM was detected. This is comparable to a previous study in Ghana that reported 8.1% carbapenem-resistant *Acinetobacter baumannii* harbouring *bla*NDM [13]. Similar studies in Egypt and Kenya have equally reported a prevalence of 19.2% and 6.25% *bla*NDM in MBL-producing *Acinetobacter* [50, 51]. The nondetection of *bla*NDM in 91.9% of the non-MBL producing *Acinetobacter* isolates in the present study may be due to the presence of other resistance genes such as *bla*GIM or *bla*SIM [21]. The MBL encoding gene (*bla*NDM) was detected mostly from wound swabs and urine. This is consistent with an earlier study in Ghana [13]. The occurrence of *bla*NDM gene may be suggestive that the *bla*NDM gene is one of the common carbapenemase genes circulating among *Acinetobacter* spp. and other related Gram-negative bacilli in Ghanaian hospitals. Metallo-beta-lactamase encoding gene (*bla*NDM) was first identified in a Swedish patient, who was hospitalized in India [52]. The Indian and Pakistan regions have been found to be the primary reserviour for *bla*NDM genes; since this discovery, *bla*NDM genes have disseminated to over 40 countries including Kenya, South Africa, Morrocco, Algeria, Iraq, Kuwait, Oman, Israel, the United Kingdom, and the United States [7]. Previously, dissemination of NDM genes was initially attributed to medical tourisms to the India subcontinent [53]. However, recent findings have associated the presence of the *bla*NDM gene to local spread in the environment [54, 55]. Metallo-beta-lactamase encoding *bla*NDM gene presently is a public health menace and infections caused by bacteria carrying these genes are difficult to treat. Furthermore, they have a high propensity for horizontal transfer to neighboring Gram-negative bacilli; hence, the presence of *bla*NDM gene in Ghanaian hospital is worrisome and calls for prompt detection, surveillance, and strict infection control measures. In conclusion, PCR analysis for *bla*VIM, *bla*IMP, and *bla*NDM showed that less than 9% of 87 *Acinetobacter* spp. harboured *bla*NDM encoding genes. Also, high levels of resistance to multiple antibiotics were found amongst MBL-producing *Acinetobacter* isolates. The detection of *bla*NDM amongst MBL-producing-*Acinetobacter* is a cause for concern; therefore, strict antibiotic usage plan and infection control measures are required to prevent the spread of these resistance genes.

### 4.4. Strength and Weakness

The sample size was small and represents less than 5% of the total population of patients visiting the health facility (KBTH). This study is the second of its kind in the facility, and we were restricted by the inability to assess other isolates from previous years. A bigger multisectorial survey isolates from patients in major regional hospitals that may incorporate treatment, screening of entire panels of OXA, and MBL encoding genes and other clinical variables are planned pending appropriate funding. Access to medical history was limited; hence, our inability to link our findings to any clinical conditions. Also, there was no follow-up of patients to determine whether the treatment was administered and whether it had been effective. Furthermore, the small number of the isolates where the gene was detected makes it hard to draw solid conclusions.

## Figures and Tables

**Table 1:** Primers used for the detection of MBL genes.

Primer name	Primer sequences	Genes	Size (bp)	Reference
IMP-R	GGAATAGAGTGGCTTAACTCTC	*bla*IMP	232	[24]
IMP-F	GTTTAACAAAACAACCACC			
VIM-R	TGGTGTTTGGTCGCAAT	*bla*VIM	390	[24]
VIM-F	CGAATGCGCAGCACCAG			
NDM-R	CGGAATGGCTCATCACGATC	*bla*NDM	621	[24]
NDM-F	GGTTTGGCGATCTGGTTTTC			

**Table 2:** Age group, sex ,and clinical specimen distribution among *Acinetobacter* spp.

Sex	Age groups							
	<1	1-10	11-20	21-30	31-40	41-50	> 50	TOTAL (%)
Female	11	0	6	13	8	3	13	54 (62.1)
Male	5	7	0	4	1	4	12	33 (37.9)
Total	**16**	**7**	**6**	**17**	**9**	**7**	**25**	**87 (100)**
Specimen								
Aspirate	1	—	—	2	—	—	—	3 (3.5)
Ear swab	5	—	—	1	—	1	2	8 (9.2)
Eye swab	5	—	—	—	—	—	—	6 (6.9)
Urine	1	1	2	6	6	1	8	25 (28.7)
Wound swab	4	6	4	8	3	5	15	45 (51.7)
Total	**16**	**7**	**6**	**17**	**9**	**7**	**25**	**87 (100)**

**Table 3:** Resistant pattern of *Acinetobacter* isolates.

Antibiotics		CDT-ve	CDT-ve	*p* value
	Resistant	Isolates	Isolates	
	No. (%)	(23) (%)	(29) (%)	
AMP	82 (94.3)	23 (100)	27 (93.1)	0.497
AMC	79 (90.8)	22 (95.7)	27 (93.1)	1
AMK	22 (25.3)	8 (34.8)	8 (27.6)	0.763
CTX	75 (86.2)	23 (100)	27 (93.1)	0.497
CXM	79 (90.8)	23 (100)	27 (93.1)	0.497
CAZ	66 (75.9)	23 (100)	29 (100)	1
COT	61 (70.1)	17 (73.9)	21 (72.4)	1
CIP	56 (64.4)	20 (87.0)	23 (79.3)	0.714
GEN	63 (72.4)	21 (95.7)	27 (93.1)	0.159
MER	52 (59.8)	23 (100)	29 (100)	1
NIT	23 (92.0)	14 (100)	7 (77.8)	0.261
LEV	59 (67.8)	22 (95.7)	24 (82.7)	0.21

Key: AMP: ampicillin; AMC: amoxicillin-clavulanate; AMK: amikacin; CTX: cefotaxime; CXM: cefuroxime, CAZ: ceftazidime, CIP: ciprofloxacin, COT: cotrimoxazole, GEN: gentamicin, MER: meropenem, NIT: nitrofurantoin, LEV: levofloxacin. CDT-ve: combined disc test negative; CDT+ve: combined disc test positive.

**Table 4:** Prevalence of *bla*NDM among specimens, age, sex, and CDT.

Variables	CDT+ve	CDT-ve	PCR	*p* value
	(*n* = 23)	(*n* = 29)	*bla*NDM	
	No. (%)	No. (%)	No. (%)	
Sex				
Female	15 (65.2)	15 (51.72)	4 (57.1)	0.4026
Male	8 (34.8)	14 (48.3)	3 (42.9)	0.4026
Specimen				
Aspirate	—	3 (10.34)	—	0.2455
Eye swab	—	—	—	
Ear swab	3 (13.04)	1 (3.44)	1 (14.3)	0.3101
Wound swab	6 (26.1)	16 (55.2)	4 (57.1)	0.0493∗
Urine	14 (60.9)	9 (31.0)	2 (28.6)	0.0491∗
Age group (years)				
<1	3 (13.04)	3 (10.34)	3 (42.9)	1
1-10	2 (8.7)	4 (13.8)	—	0.6821
11-20	1 (4.4)	—	—	0.4423
21-30	3 (13.0)	7 (24.14)	1 (14.3)	0.4815
31-40	5 (21.7)	3 (10.34)	1 (14.3)	0.4411
41-50	—	2 (6.9)	—	0.497
>50	9 (39.1)	10 (34.5)	2 (28.6)	0.778

## Data Availability

The datasets used and/or analysed during the current study are available from the corresponding author on reasonable request.
